# Rapid compensatory changes in the expression of EAAT-3 and GAT-1 transporters during seizures in cells of the CA1 and dentate gyrus

**DOI:** 10.1186/1423-0127-19-78

**Published:** 2012-08-29

**Authors:** Laura Medina-Ceja, Flavio Sandoval-García, Alberto Morales-Villagrán, Silvia J López-Pérez

**Affiliations:** 1 Laboratorio de Neurofisiología y Neuroquímica, Departamento de Biología Celular y Molecular, Centro Universitario de Ciencias Biológicas y Agropecuarias, Universidad de Guadalajara, Km. 15.5 Carretera Guadalajara-Nogales Predio “Las Agujas”; Nextipac, Zapopan, Jalisco, CP 45110, Mexico

**Keywords:** 4-Aminopyridine, EAAT-3, GAT-1, Hippocampus, Immunofluorescence, Seizures

## Abstract

**Background:**

Epilepsy is a neurological disorder produced by an imbalance between excitatory and inhibitory neurotransmission, in which transporters of both glutamate and GABA have been implicated. Hence, at different times after local administration of the convulsive drug 4-aminopyridine (4-AP) we analyzed the expression of EAAT-3 and GAT-1 transporter proteins in cells of the CA1 and dentate gyrus.

**Methods:**

Dual immunofluorescence was used to detect the co-localization of transporters and a neuronal marker. In parallel, EEG recordings were performed and convulsive behavior was rated using a modified Racine Scale.

**Results:**

By 60 min after 4-AP injection, EAAT-3/NeuN co-labelling had increased in dentate granule cells and decreased in CA1 pyramidal cells. In the latter, this decrease persisted for up to 180 min after 4-AP administration. In both the DG and CA1, the number of GAT-1 labeled cells increased 60 min after 4-AP administration, although by 180 min GAT-1 labeled cells decreased in the DG alone. The increase in EAAT-3/NeuN colabelling in DG was correlated with maximum epileptiform activity and convulsive behavior.

**Conclusions:**

These findings suggest that a compensatory mechanism exists to protect against acute seizures induced by 4-AP, whereby EAAT-3/NeuN cells is rapidly up regulated in order to enhance the removal of glutamate from the extrasynaptic space, and attenuating seizure activity.

## Background

Epilepsy is a neurological disease with a lifetime prevalence of 2-5% (excluding febrile seizures), affecting approximately 67 million people worldwide [1]. Epilepsy is thought to reflect an imbalance between excitatory and inhibitory neurotransmission [2,3] and indeed increased levels of glutamate, the principal excitatory neurotransmitter in the central nervous system (CNS), have been well documented during seizures [2,4]. This excess glutamate must be removed from the synaptic space by membrane proteins called transporters. At least five subtypes of glutamate transporters have been described, along with several variants: GLAST (EAAT-1), GLT-1 (EAAT-2, EAAT-2a, EAAT-2b and EAAT-2c), EAAC-1(EAAT-3), EAAT-4 and EAAT-5 [5-7]. Glutamate transporters are expressed by neurons and glial cells in many regions of the brain and for example, the EAAT-3 transporter is expressed in the dendrites and soma of granule and pyramidal cells of the hippocampal dentate gyrus and CA1 region, respectively. Moreover, EAAT-3 transporters are found at both asymmetric and symmetric synapses [8-10]. In conjunction with the cysteine/glutamate antiporter X_c_^-^, EAAT-3 protects neuronal HT22 cells (an immortalized hippocampal cell line) from oxidative glutamate toxicity [11]. Indeed, altered EAAT-3 expression has been described in epilepsy and in response to particular seizure types. Accordingly, in a pilocarpine-induced rat model of Temporal Lobe Epilepsy (TLE), EAAT-3 gene and protein expression increases rapidly in dentate granule cells in association with long-lasting epilepsy [12]. Similar findings have been reported in TLE patients [13-15], in whom an increase in EAAT-3 protein levels and in the percentage of EAAT-3-IR neurons occurs in CA2 and in the granule cell layer of the dentate gyrus.

GABA (gamma-aminobutyric acid), the principal inhibitory neurotransmitter, is also thought to play an important role in epilepsy and four GABA transporter subtypes have been identified in mammals: GAT-1, GAT-2, GAT-3 and GAT-4 [16]. In particular, GAT-1 is expressed in the pyramidal and granule cell layers of the hippocampal formation, and this transporter is considered therapeutically significant given the antiepileptic effects elicited by its blockade [17,18]. Changes in GAT-1 protein and mRNA expression have also been described in various epilepsy models, including the kainic acid, picrotoxin, FeCl_2_ models, and in TLE patients [13,19-21].

Here we have studied the changes in EAAT-3 and GAT-1 transporter protein expression in hippocampal cells of the dentate gyrus (DG) and CA1 at different times after 4-aminopyridine (4-AP) administration because it was demonstrated that 4-AP increases the levels of glutamate and GABA in the hippocampal extracellular space, as well as glutamate produces hyperactivation of its receptors and seizures [2,3,22]. Although, participation of glutamate and GABA transporters in seizures is well documented, there is no evidence about their contribution in this model of seizures considering their possible effect in extracellular neurotransmitters levels observed previously and relation with EEG activity as well as convulsive behavior. For this reason, we used dual immunofluorescence to determine whether glutamate or GABA transporters co-localize with the neuronal marker NeuN. In parallel, we recorded EEG activity and analyzed seizure behavior using a modified version of the Racine Scale [23] to investigate the correlation between seizure intensity and changes in EAAT-3/Neun or GAT-1/Neun co-labeled cells. In addition, we chose the entorhinal cortex (EC) in order to inject 4-AP because it produces an epileptiform pattern in EEG recordings previously studied in our laboratory [2,23,24]. Our results show a rapid compensatory effect in the average number of cells immunolabelled for EAAT-3 during maximum epileptiform activity associated with a high convulsive behavior in bilateral DG region. We conclude that this compensatory mechanism has the purpose to increase glutamate clearance from the extrasynaptic space due to the raise in glutamate levels induced by K^+^ channel blocked effect of 4-AP. Also, increases in GAT-1/Neun labeled DG cells observed may facilitate increased GABA release in this region via reverse transport, in order to enhance inhibitory effect in response to the excitatory stimuli produced by seizures and the effect of glutamate exposure in 4-AP treated rats, but it is necessary additional experimental work to confirm this hypothesis.

## Methods

### Animal surgery

Twenty-four adult male Wistar rats (250–350 g in weight) were used in the present study. All experimental procedures were designed in order to minimize animal suffering and the total number of animals used. All rats were maintained in individual cages in a temperature-controlled room, on a 12-h light/dark cycle with *ad libitum* access to food and water. This protocol was conformed to the Rules for Research in Health Matters (Mexican Official Norms NOM-062-ZOO-1999, NOM-033-ZOO-1995) and it was approved by the local Animal Care Committee.

Rats were first anesthetized with isofluorane in 100% O_2_ and secured in a Stoelting stereotaxic frame with the incisor bar positioned at −3.3 mm. A stainless steel guide cannula (0.5 mm internal diameter) was implanted into each rat through a hole drilled in the skull, and it was positioned in the right entorhinal cortex (rEC) at the following stereotaxic coordinates relative to bregma (rEC: AP −8 mm, L 4.6 mm, V 4 mm). This cannula was used to insert an injection needle (V 5 mm) that also served as a recording electrode. The cannula was insulated by varnishing the entire surface except a 1 mm portion of its tip. Four stainless steel screws were attached to the skull, two above bregma and two above the cerebellum, which served as indifferent and ground electrodes, respectively. Three surface electrodes with the same characteristics as the electrodes described above were implanted into the skull above: the left occipital cortex binocular (lOCB, AP −8 mm, L −4.6 mm relative to bregma); and the right and left occipital cortex (rOC/lOC, AP −5.6 mm, L 5.0 mm; AP −5.6 mm, L −5 m relative to bregma). The guide cannula and surface electrode wires were attached to a socket connector and fixed to the skull with acrylic dental cement.

### Drug administration and EEG recording

After surgery, the animals were allowed to recover for 24 h and they were then divided into experimental groups as follows: 3 control groups (n = 3 per group) received an injection of the vehicle alone (NaCl, 0.9%) and 30, 60 or 180 min after injection the animals were sacrificed; 3 experimental groups of animals (n = 5 per group) were injected with 10 nmols 4-AP, and sacrificed similarly 30, 60 or 180 min after injection.

4-AP (Sigma St. Louis, MO, USA) was diluted in the appropriate concentration of NaCl to maintain iso-osmolarity. Both vehicle and 4-AP solutions were injected locally into the rEC at a flow rate of 0.5 μl/min for 2 min (final volume 1 μl) using a microsyringe mounted on a BAS microinjection pump.

The rats were placed in a container unit and the electrodes connected to a cable fixed to a balanced arm. EEG activity was recorded using a Grass polygraph model 6 with a low-frequency filter at 1 Hz and a high frequency filter at 300 Hz, and it was sampled at 100 Hz/channel (four channels). Data was stored on a computer hard disk and analyzed with AcqKnowledge software from Biopac Systems MP150 (Biopac Systems, Inc. Goleta, CA, USA). After recording basal activity for 30 min, vehicle or 4-AP (10 nmol) was administered and the animals were then observed continuously for 30, 60 or 180 min, during which time EEG activity was recorded. The basal electrical activity of each group was analyzed, and the amplitude and frequency averaged over a 5 min recording period. The data from the experimental groups was analyzed by measuring the amplitude of single epileptiform discharges over a 5 min period of the recording at different times after drug administration. The frequency of the EEG epileptiform activity (the number of single epileptiform discharges during a 1 sec seizure period) was averaged manually for 5 min according to the different time periods studied. Propagation of this epileptiform activity to other areas was also analyzed.

### Behavioral study

Animal behavior was scored by continuous observation before, during and after vehicle or drug administration, using a modified version of the Racine scale [23]. Briefly, behavior was scored as follows: 0, behavioral arrest (motionless), piloerection, excitement and rapid breathing; 1, movement of the mouth, lips tongue and vibrissae, salivation; 2, head and eye clonus; 3, forelimb clonus, “wet dog shakes”; 4, clonic rearing; 5, clonic rearing with loss of postural control and uncontrolled jumping.

### Perfusion and Immunofluorescence

Rats were anaesthetized with nembutal (60 mg/kg, i.p.) and transcardially perfused with phosphate buffer saline (PBS, 0.1 M, pH 7.4, 37 °C), followed by 4% paraformaldehyde (in 0.1 M PBS, pH 7.4) containing 0.1% glutaraldehyde. The animal’s brain was removed and post-fixed for 12 to 16 h at 4 °C. The brains were then mounted on a vibratome (Leica, VT1000S, Germany) and sliced coronally at a thickness of 50 μm. These sections were collected consecutively in separate wells of an incubation chamber containing PBS.

Antibodies directed against the EAAT-3 glutamate transporter (rabbit polyclonal antiserum, c-terminal amino acids 455–524 of human EAAT-3: Santa Cruz Biotechnology, Inc., Santa Cruz, CA) and the GAT-1 GABA transporter (rabbit polyclonal antiserum, c-terminal amino acids 588–599 of rat GAT-1: Chemicon International, Temecula, CA) were used. A monoclonal NeuN antiserum (Chemicon International) was used to identify neurons.

Immunofluorescence was performed as described previously [25]. Briefly, EAAT-3 and GAT-1 immunofluorescence was performed using PBS and TRIS-buffer saline (TBS; 0.1 M, pH 7.4), respectively, in conjunction with the NeuN antibody. Tissue sections were washed twice for 15 min in PBS or TBS with agitation and they were then incubated for 2 h at room temperature with 5% normal goat serum (NGS) in PBS or TBS. After two 15 min washes, the sections were incubated for 72 h at 2-8°C with combinations of the primary antibodies (anti-EAAT-3, 1:500; anti-GAT1, 1:250; and anti-NeuN, 1:1000) diluted in PBS or TBS containing 5% NGS and 0.3% Triton X-100. The sections were then washed five times with agitation for 10 min in PBS or TBS and incubated with the secondary antibody with agitation for 2 h in darkness at room temperature. EAAT-3 and GAT1 were detected using goat anti-rabbit IgG-Alexa 594 (1:500: Invitrogen, Oregon, USA) in PBS or TBS containing 5% NGS, respectively, while NeuN was identified using a goat anti-mouse IgG-Alexa 488 (1:500: Invitrogen, Oregon, USA). Sections were washed five times with agitation for 15 min each in PBS or TBS [8,18] before mounting on glass slides and coverslipping using vectashield to preserve fluorescence. The sections were examined by fluorescence microscopy (Olympus, U-LH100HG, Japan) and analyzed using Photoshop CS4 software. Control sections were incubated without primary antibody or by replacing the primary antibody with normal rabbit serum, and no fluorescent signal was observed in any of the control sections (data not shown).

### Cell counting

As described previously [25], the number of cells was calculated by manual observation of fluorescence microscopy images using Photoshop CS4 software (Version 11.0). Cells of the DG or CA1 region of the hippocampus were counted in merged images of double-labelled sections (EAAT-3/NeuN or GAT-1/NeuN) using a 40x objective (5 images for each transporter in each animal). Counting was restricted to a defined area of tissue (DG or CA1) using a modification of West’s method [26]. Briefly, a number of slices at uniform intervals were selected from the total number of slices obtained per region (5 slices were selected from a total of 10 per animal) and the total number of co-labelled neurons in the DG or CA1 was counted using an optical dissector (height = 0.01 mm). This systematic scanning was performed in 50 μm slices over a defined region of interest with an area of 35,200 μm^2^ (based on the objective used and the image area captured). The number of cells counted in the optical dissector was then corrected according to the counting area, the proportion of the slices analysed (50%) and the thickness of the slice, providing a total value for the region of interest.

### Histological evaluation

To verify the location of the guide cannula in each experiment, some coronal brain sections (50 mm thick) were taken from rats previously perfused to immunofluorescence and belonging to different experimental and control groups, then stained with cresyl violet and if the cannula was implanted incorrectly the animals were excluded from the study.

### Data analysis

Comparative analysis of the experimental and control groups was performed using a one-way analysis of variance (ANOVA) and a Tukey-Kramer post hoc test. A paired Student´s *t* test was used to compare control and experimental groups at equivalent time points. The Mtab13 statistical software package was used for all analyses. Results were considered statistically significant at p < 0.05.

## Results

### EEG activity and seizure behavior in animals

**Figure 1:**
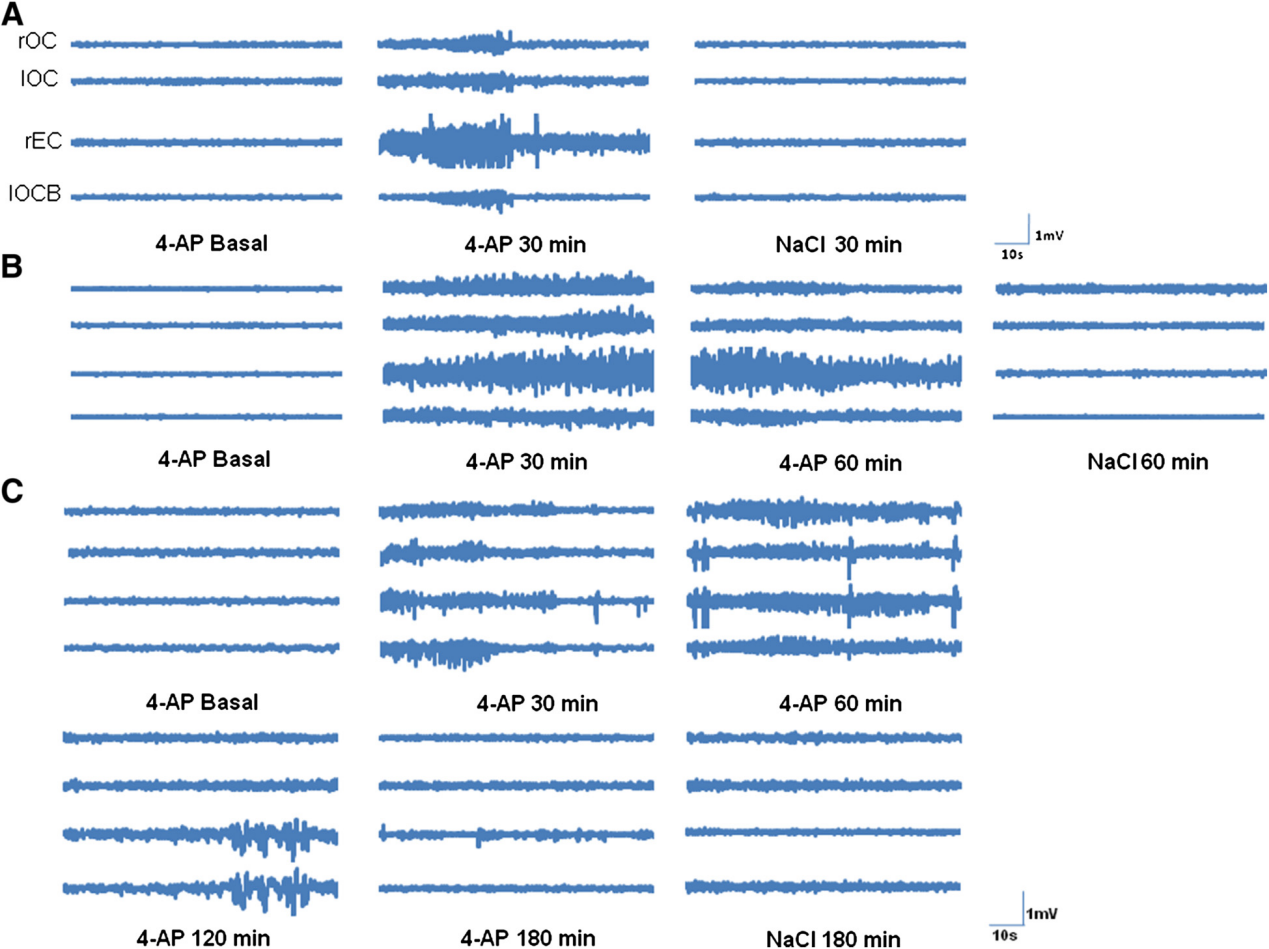
**Representative EEG recordings at different time-points after vehicle (NaCl) or 4-AP administration.A**) Left to right; EEG activity in a 4-AP rat before and 30 min after 4-AP administration, and in a control rat 30 min after NaCl administration. **B**) Left to right; EEG activity in a 4-AP rat before, 30 and 60 min after 4-AP administration, and in a control rat 60 min after NaCl administration. **C**) Left to right; EEG activity in a 4-AP rat before, 30, 60, 120, and 180 min after 4-AP administration, and in a control rat 180 min after NaCl administration. Abbreviations: right and left occipital cortex (rOC/lOC), right entorhinal cortex (rEC) and left occipital cortex binocular (lOCB).

**Figure 2:**
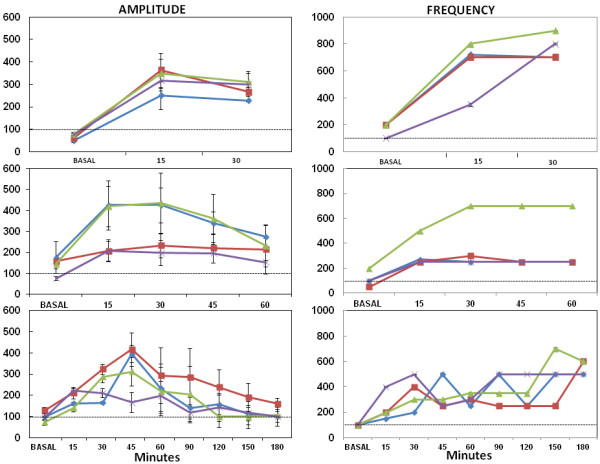
**Changes in the amplitude and frequency of EEG activity after 4-AP injection.** Graphs represent the three experimental groups of animals sacrificed at 30 (graphs in top), 60 (graphs in middle) and 180 (graphs in bottom) min after 4-AP administration. The time course is represented on the x-axis, while the y-axis represents the data as percentage of change with respect to electrical basal activity (100%) of 24 control animals. Error bars represent SEM. Abbreviations: right and left occipital cortex (blue diamond rOC / red square lOC), right entorhinal cortex (green triangle rEC) and left occipital cortex binocular (violet asterisk lOCB).

In vehicle-treated animals, EEG activity was characterized by the presence of slow physiological waves of low amplitude and frequency, similar to the basal EEG activity in the animals administered 4-AP (Figures 1 and 2). No convulsive behavior was observed in the control animals that received the vehicle alone, and they displayed only sporadic tidying and scratching behavior before and after NaCl administration. 4-AP administration to the rEC (n = 15) induced epileptiform activity, which was characterized by the presence of trains of poly-spikes and low amplitude and frequency spike-wave complexes that became more intense during the course of the experiment. The first epileptiform discharge was observed in the rEC (134 ± 86.4 s), and then in the lOC (151 ± 79.5 s), lOCB (263 ± 136 s) and finally in the rOC (266 ±136 s).

The maximum amplitude and frequency of epileptiform activity was observed between 30 and 60 min after 4-AP administration in animals sacrificed at 180 min, and it was associated with a score of 3–4 on the modified Racine Scale [23] (Table 1). In this experimental group, epileptiform activity decreased in amplitude, particularly between 90 and 120 min after 4-AP administration, returning to basal levels 180 min after 4-AP administration (Figures 1 and 2). These animals received scores of 1, 2 or 3 on the modified Racine scale at the end of experiment.

**Table 1:** Seizure behavior of the three groups of rats following intra-rEC administration of 4-AP (10 nmol, n = 5 per group)

**Behavior after 4 AP Injection**						
**RAT**	**Minutes**					
	**15**	**30**	**45**	**60**	**120**	**180**
**1**–**5** (A)	0/1/2/3	0/1/2/3				
**6**–**10** (B)	0/1/2	0/1/2/3/4	0/1/2/3/4	0/1/2/3		
**11**–**15** (C)	0/1/2/3	1/2/3/4	0/1/2/3/4	0/1/2/3/4	0/1/2/3	0/1/2/3

The values represent seizure behavior according to the modified Racine Scale [23]: 0, behavioral arrest (motionless), piloerection, excitement and rapid breathing; 1, movement of the mouth, lips tongue and vibrissae, salivation; 2, head clonus and eye clonus; 3, forelimb clonus, “wet dog shakes”; 4, clonic rearing; 5, clonic rearing with loss of postural control and uncontrolled jumping.

The rats were sacrificed 30 (A), 60 (B) or 180 (C) min after 4-AP injection.

### Effect of 4-AP on EAAT-3 colabelling

**Figure 3:**
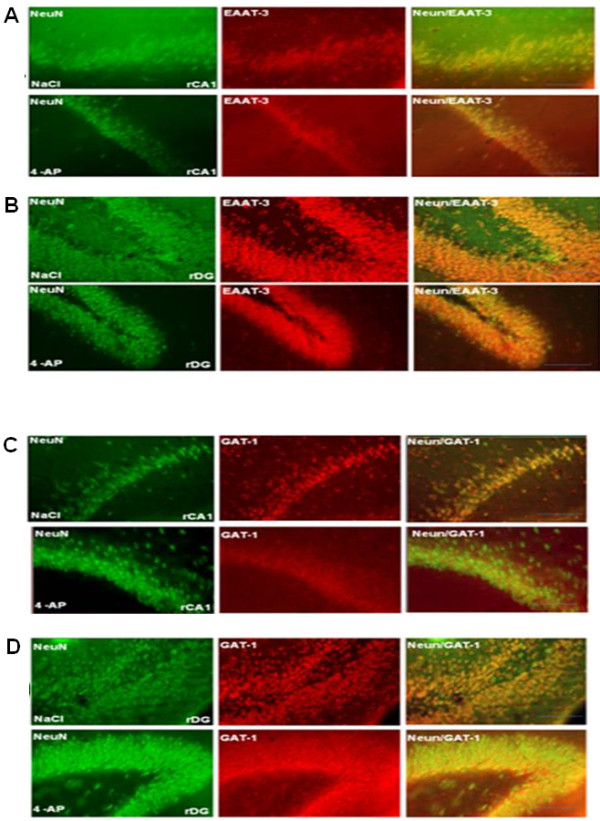
**Representative fluorescence microscopy images obtained from control and experimental animals where it is observed NeuN, EAAT-3 or GAT-1 labeled cells, as well as co-labeled cells (NeuN/EAAT-3 or NeuN/GAT-1) in various brain regions: A and C) in right CA1 (rCA1); B and D) in right dentate gyrus (rDG).** Scale bar = 50 μm.

**Figure 4:**
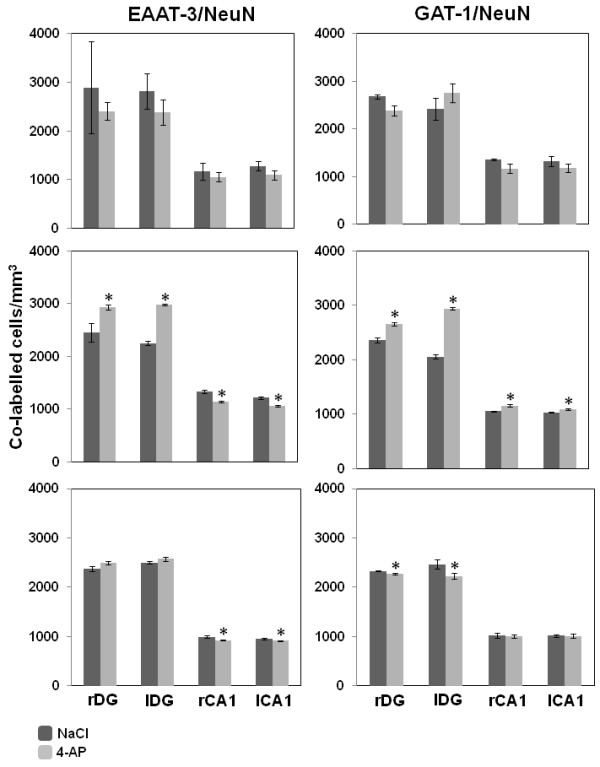
**Number of co-labelled cells per mm**^**3**^**in the different brain regions of animals sacrificed 30 (top), 60 (middle) and 180 (bottom) min after vehicle or 4-AP administration.** Abbreviations as in Figure 3.

EAAT-3 labeling was observed around the soma and dendrites of granule cells in the DG and pyramidal cells in the CA region, both in control and experimental animals (Figure 3). There were no significant differences in the number in EAAT-3/NeuN dual labeled cells observed in experimental and control animals sacrificed 30 min after injection (Figure 4). However, more EAAT-3/NeuN dual labeled cells were evident in the DG of animals sacrificed 60 min post 4-AP injection than in control animals (right DG (rDG), 2931 ± 52 vs. 2457 ± 178; left DG (lDG), 2980 ± 14 vs. 2253 ± 42 co-labeled cells/mm^3^, p<0.001) that represent 19% and 32% of change in the rDG and lDG, respectively. In parallel, we observed a decrease in the number of EAAT-3/NeuN co-labeled cells in the CA1 of 4-AP-treated animals when compared with controls (right CA1 (rCA1), 1145 ± 18 vs.1327 ± 31; left CA1 (lCA1), 1059 ± 16 vs. 1217 ± 21 co-labeled cells/mm^3^, p<0.001), this is 14% and 13% of change in the rCA1 and lCA1, respectively. In animals sacrificed 180 min after 4-AP injection, the number of CA1 pyramidal cells expressing EAAT-3 decreased when compared with the control animals (rCA1, 927 ± 14 vs. 992 ± 23; lCA1, 915 ± 11 vs. 956 ± 17 co-labeled cells/mm^3^, p<0.05), that represent 7% of change in rCA1 and 5% in lCA1. By contrast, no significant changes were observed in the DG region.

### Effect of 4-AP on GAT-1 colabelling

GAT-1 labeling was observed around the soma of granule cells in the DG and pyramidal cells in CA1, both in control and 4-AP-treated animals (Figure 3). No significant changes in GAT-1/NeuN colabelling were observed in either region between control and 4-AP-treated animals sacrificed at 30 min (Figure 4). However, 60 min after 4-AP administration we observed an increase in the number of GAT-1 labeled cells in the DG and CA1 when compared with vehicle-treated animals (rDG, 2656 ± 36 vs. 2364 ± 42; lDG, 2949 ± 24 vs. 2058 ± 44; rCA1, 1155 ± 23 vs. 1055 ± 6; lCA1 1090 ± 11 vs. 1032 ± 10 co-labeled cell/mm^3^, p<0.05), this is 12% and 43% of change in rDG and lDG, respectively, while in rCA1 and lCA1 was observed 9% and 5% of change. In animals sacrificed 180 min after 4-AP injection, the number of GAT-1 labeled cells in the DG decreased when compared with vehicle-treated animals (rDG, 2270 ± 19 vs. 2329 ± 8; lDG, 2224 ± 60 vs. 2468 ± 87 co-labeled cells/mm^3^, p<0.05), that represent 3% and 10% of change in rDG and lDG, respectively, while there were no changes in GAT-1 in the CA1.

## Discussion

Local administration of 4-AP into the rEC induced epileptiform activity characterized by poly-spike trains and spike-wave complexes, peaking between 30 and 60 min after 4-AP administration, and consistent with previous studies in our laboratory [23,24]. Moreover, the maximal EEG activity coincided with convulsive behavior that was associated with a score of 3/4 on the modified Racine Scale [23]. Epileptiform discharges were first detected in the rEC, followed by the lOC and lOCB, and finally in the rOC.

EAAT-3 colabelling was detected in the soma and dendrites of dentate granule cells and pyramidal cells of control groups. In agreement with our study, Holmseth et al. [27] detected EAAT-3 labeling intracellularly in soma and dendrites in DG of hippocampus, they estimated the mean EAAT-3 density (90 molecules/μm^2^) in dendritic membrane but the most of the EAAT-3 was located intracellularly assuming that EAAT-3 is evenly distributed. Animals sacrificed 30 min after 4-AP administration exhibited no changes in the average number of cells immunolabelled for EAAT-3 or GAT-1, in either the CA1 or DG. However, 60 min after 4-AP-induced epileptiform activity, the number of EAAT-3/NeuN co-labeled cells increased bilaterally in the DG while decreased in CA1. The present findings are supported by early studies describing decreased EAAT-3 immunolabeling in rat pyramidal cells of CA1 just 4 h after seizure onset produced by kainic acid administration [28]. Increased EAAT-3 immunoreactivity (IR) in granule cells of DG during the acute epileptic phase (24 h) was observed in the animal model of status epilepticus (SE) induced by electrical stimulation [29], and increases in EAAT-3 and GAT-1 IR have been described in granular and pyramidal cells of hippocampal sections from TLE patients [13,14]. Although, these last data cannot be directly compared with the present results, all these findings indicate important changes in expression of EAAT-3 transporter during seizures and epilepsy. The decreases in CA1 EAAT-3 labeled cells described here contrast with the increases in EAAT-3 IR neurons in the CA1-3 of animals with SE reported previously [29]. This discrepancy may be due to differences in the protocols used to induce seizures, in the EAAT-3 antibodies used and in the time between seizure induction and tissue processing, further increasing the potential divergence in the results.

CA1 and CA3 neurons have already been described to be more susceptible to seizures than those in other CA regions [30,31] and neuronal damage in CA1/CA3 was observed after 4-AP injection in hippocampus [32] that contrast with abundant NMDA receptors found in this region [33], similarly antagonists to this receptor protects against seizures and damage [34,35]. These early studies support our results about decreased EAAT-3 labeled cells observed in CA1. Indeed, kainic acid administration decreases EAAT-3 IR and EAAT-3 mRNA expression in CA1 pyramidal cells [28]. Together, these findings suggest a rapid down regulation of EAAT-3 transporter expression in the CA1 during seizures.

Considering that only 20-30% of EAAT-3 is localized at the plasma membrane [36,37] and the intracellular EAAT-3 can be inserted rapidly into the plasma membrane in response to activity or pathological conditions like seizures [36,38,39], the increase of EAAT-3 in soma of granule cells found 60 min after seizures induced by 4-AP could function as a available pool of EAAT-3 for redistribution to the plasma membrane to facilitates the capture as well as the clearance of glutamate excess from the extrasynaptic space, particularly given the localization of EAAT-3 transporters in asymmetrical and symmetrical synapses in the soma and dendrites of granule cells [8,10]. This hypothesis is supported by our previous studies where an increase in extracellular glutamate levels (up 400% of change with respect to the baseline) in hippocampus was observed during the first epileptiform discharge after 4-AP (10 nmol) injection into the EC, these increases in glutamate levels were decreased as the seizure progress and at 60 min after 4-AP administration, glutamate concentrations were returned to basal condition, this temporal profile agree with the results obtained here [4,40]. In addition, the methodologies used in these studies (an electrochemical biosensor and enzymatic reactor with electrochemical detection) allowed us to improve time resolution of about 1 min per microdialysis sample [4,40]. Also, co-expression of the NMDA glutamate receptor with the EAAT-3 transporter in Xenopus oocytes decreases the activation of the former [41], suggesting a protective role of the EAAT-3 transporter in excitotoxic conditions and in co-operation with the cysteine/glutamate antiporter X_c_^-^, the EAAT-3 transporter helps to protect neuronal HT22 cells (an immortalized hippocampal cell line) from oxidative glutamate toxicity [11]. Increases in EAAT-3/NeuN colabelling in dentate granular cells may also enhance inhibitory activity by increasing GABA synthesis. In agreement, the GAD/GABA markers in granule cells are transiently up-regulated in response to seizures [42,43]. The EAAT-3 transporter also participates in the rapid adaptation of pre-synaptic inhibitory terminals to alterations in local network activity by replenishing vesicular glutamate content, which can then be converted to GABA for release [44,45]. Furthermore, kindled seizures induce GABAergic fast synaptic inhibition in the mossy fibers of the DG to CA3 system [46].

Animals sacrificed 180 min after 4-AP injection exhibited bilateral decreases in the average number of EAAT-3/NeuN co-labeled pyramidal cells in the CA1. Decreased EAAT-3 IR was also described previously in rat CA1 pyramidal cells 4 hours after KA treatment [28] and diminished EAAT-3 mRNA expression was reported in the rat hippocampus 4 h after ferric chloride treatment [47]. Taken together, these results suggest a rapid down regulation of EAAT-3 transporter during seizures induced by diverse chemical agents. The decrease in the average number of EAAT-3/NeuN co-labeled pyramidal cells from 60 to 180 min after 4-AP administration supports the hypothesis that CA1 neurons are highly susceptible to seizures. However, decreased EAAT-3 transporter expression can induce seizures, as seen when using an anti-sense oligopeptide antibody to EAAT-3 [48].

In the present study, maximum seizure intensity and convulsive behavior elicited by a low dose of 4-AP (10 nmols) was correlated with a rapid up-regulation of EAAT-3/NeuN colabelling 60 min after 4-AP injection. It is established that 4-AP increase extracellular levels of glutamate that originate from pre-synaptic nerve endings to produce an overactivation of glutamate receptors and subsequent seizures as well as neurodegeneration of the CA1, CA2 and CA3 hippocampal subfields [2,3] This effect is blocked by glutamate receptor antagonists of both the NMDA and the non-NMDA types in vivo as in vitro experiments [34,49]. Also it is known that CA1 contains a high expression of NMDA receptors [50]. EAAT-3 as a neuronal transporter, not only to uptake glutamate from synaptic cleft to maintain of low extracellular glutamate, also it contributes to multiple aspects of synaptic signaling: EAAT-3 limits glutamate spillover and participates to neuronal uptake of cysteine, a critical precursor for glutathione synthesis and importantly EAAT-3 can attenuate the activation of perysinaptic NMDA receptors [41,51]. With base in these arguments and as an effect caused by 4-AP, the increase in EAAT-3 colabelled cells in DG after 60 min post-seizures induced by 4-AP could function as an available pool of EAAT-3 for redistribution to the plasma membrane to facilitate the capture as well as the clearance of glutamate excess from the extrasynaptic space to attenuate the overactivation of perysinaptic NMDA receptors. This compensatory mechanism has the purpose to attenuate seizures and the associated convulsive behavior; these latter effects were observed 180 min after 4-AP injection (Figure 1C2 and Table 1).

GAT-1 colabelling was detected in the soma and dendrites of dentate granule cells and pyramidal cells, in agreement with previous studies [20,52]. Bilateral increases in the number of GAT-1/NeuN co-labeled cells in the DG and CA1 were observed in this study. These results are related with significant increases of GAT-1 mRNA found bilaterally in the hippocampal DG at 1 h after kindled generalized seizures [53]. In addition, this up-regulation of GAT-1/NeuN colabelling observed 60 min after 4-AP administration may augment GABA release into the synaptic cleft via reverse transport, counteracting the excitatory activity produced by glutamate release induced by 4-AP. This hypothesis is supported by the following experimental evidences. Reverse transport via GAT-1 transporters has been described in cultured hippocampal neurons, where it facilitates phasic inhibition through the activation of GABA-A receptors in both normal and pathological conditions [54,55]. GAT-1 reverse transport and activation of nearby GABA receptors have also been reported in glia [55,56]. Given the dual role of granule cells in GABA and glutamate neurotransmission during seizures [46] reverse transport via GAT-1 may increase GABA release from dentate granule cells, thereby reducing hyperactivity and seizure activity in this region. In addition, previous studies have demonstrated that 4-AP increase GABA levels in hippocampus at different 4-AP concentrations during and after 4-AP administration [22,57]. However, it is necessary additional experimental work using a high time resolution methodology to confirm a better relation between GABA levels, EEG activity and GAT-1 labeling throughout seizures induced by 4-AP. Finally, we observed a decrease in GAT-1/NeuN colabelling in dentate granule cells 180 min after seizure induction, which was accompanied by a decrease in seizure activity. Although, we cannot compare the decrease of GAT-1 observed 180 min after 4-AP treatment with other studies, since there are not available data in this matter, we could suggest that the rapid upregulation of GAT-1 labeled cells seen in the DG and CA1 at 60 min was sufficient to abolish epileptiform discharges in these regions as well as the seizure behavior of animals observed 180 min after 4-AP.

## Conclusion

Our results provide evidence that the average number of cells immunolabelled for EAAT-3 is rapidly up-regulated in dentate granule cells during acute seizures. We propose that this represents a protective and compensatory adaptation to enabling more rapid and efficient removal of glutamate from the extrasynaptic space induced by 4-AP and thus attenuating seizure activity. The increase in EAAT-3/NeuN colabelling may also enhance inhibitory activity in dentate granule cells by increasing GABA synthesis, further counteracting the hyperexcitation of glutamate. Also, our results reveal a high degree of seizure susceptibility in CA1 pyramidal cells, as demonstrated by a decrease in EAAT-3 expression in this region. Finally, the increase in GAT-1/NeuN colabelling observed in DG cells may facilitate increased GABA release in this region via reverse transport, further attenuating seizure activity, but it is necessary additional experimental work to confirm this hypothesis. Together, these findings contribute to our understanding of the role of glutamate and GABA transporters in acute seizures particularly induced by 4-AP.

## Competing interests

The authors have no competing interests.

## Authors’ contributions

LMC participated in supervising experiments, analysis and discussion of results, manuscript preparation and final revision; FSG contributed in experiments and analysis of results; AMV and SJLP contributed in discussion of results and final revision. All authors read and approved the final manuscript.
